# Stripy Nanoparticles Revisited

**DOI:** 10.1002/smll.201001465

**Published:** 2012-11-23

**Authors:** Yann Cesbron, Chris P Shaw, James P Birchall, Paul Free, Raphaël Lévy

**Affiliations:** Structural and Chemical Biology, Institute of Integrative Biology, University of Liverpool Biosciences BldgCrown Street, L69 7ZB, UK; CNRS, UMR 6290, Institut Génétique et Développement de Rennes, F-35043 Rennes, France, Université de Rennes 1, Université Européenne de Bretagne, Structure fédérative de recherche Biosit, Faculté de MédecineF-35043 Rennes, France; Institute of Materials Research and Engineering, A*STAR (Agency for Science, Technology and Research)3 Research Link, Singapore 117602, Singapore

In May 2004, Jackson et al. published an article entitled “Spontaneous assembly of subnanometre-ordered domains in the ligand shell of monolayer-protected nanoparticles”.^1^ This was to become the first of a series which now counts over ten research articles.^1^, ^2^ All of these are based on the existence of “stripy” nanoparticles, where the stripes are constituted by the self-organization of two different thiolated ligands. A number of unusual and exciting properties are attributed to the nanoscale organization of the ligands. Thus, stripy nanoparticles are reported as being “extremely effective in avoiding non-specific adsorption of a variety of proteins”,^1^ having the ability to “penetrate the plasma membrane without bilayer disruption”2j and having poles which are particularly reactive and can be selectively addressed to obtain divalent nanoparticles.2c This series of articles and the corresponding structure–property relationships are important because of their direct impact on our understanding of several of the key contemporary problems in the field of nanoscience. The latter include the characterization of nanomaterials with sub-nanometer resolution,^3^ the possibility of controlling the self-organization of ligands on gold nanoparticles,^4^ the understanding of nanoparticle–biomolecule and nanoparticle–cell interactions,^5^ and the intracellular delivery of nanoparticles.^6^ The proposed stripy structure is based on scanning tunneling microscopy (STM) images which have not yet been reproduced by other groups to date. Our interest lies in nanoparticle surface engineering^7^ and the interaction of nanoparticles with living cells.6b Carefully following the published results for producing stripy nanoparticles, we failed to substantiate a number of the claims made about their properties, so in the first part of this paper we critically revisit the published evidence for stripiness and in the second part we present our own results regarding their physicochemical properties.

We first consider a simple geometrical problem. An STM topography image of a spherical particle is, in first approximation, a 2D projection of the top hemisphere. If a spherical particle is covered with regularly spaced stripes, what should be the apparent width of the stripes? For a 5.8 nm-diameter sphere with 18 regularly spaced 1 nm-wide stripes (9 per hemisphere), the width of the projected stripes on a 2D image decreases rapidly as the STM tip goes from the top of the sphere to its edge, perpendicularly to the stripe direction (**Figure** **1**a). A model theoretical STM image of the 5.8 nm stripy nanoparticle is constructed (Figure 1b) and a theoretical line profile of it is shown (Figure 1c).

**Figure 1:**
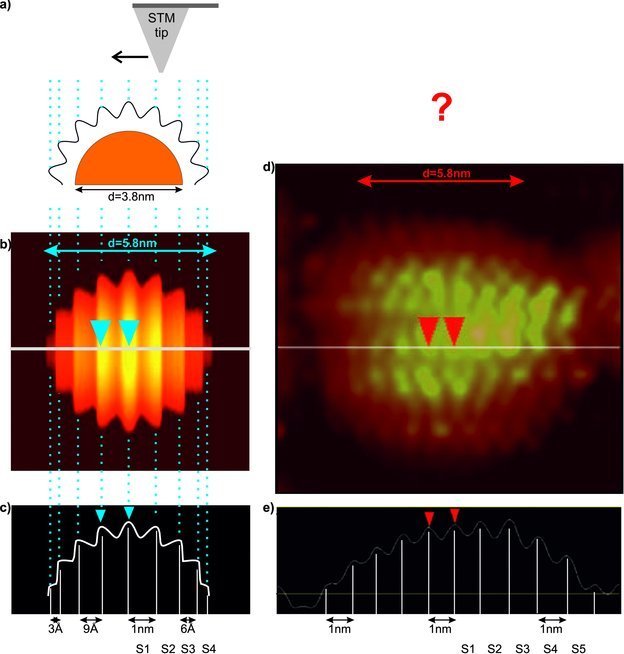
Comparison of the predicted and reported stripe widths for a 5.8 nm diameter nanoparticle. a) STM tip scanning over a stripy nanoparticle perpendicularly to the stripe orientation (section view); b) predicted STM image with color coding showing the predicted height at each position; c) height profile corresponding to the white line in (b); d) experimental STM image (adapted from Figure 1b of Jackson et al.^1^); e) Height profile corresponding to the white line in image (d), (adapted from Figure 1d of Jackson et al.^1^). Panels (d) and (e) were adapted with permission.^1^ Copyright 2004, Macmillan Publishers Ltd.

We now compare this model with Jackson et al.’s experimental results.^1^ An exemplary STM image of a nanoparticle from their manuscript is shown in Figure 1d. The diameter of the gold core was measured by the authors as being 3.8 nm (by transmission electron microscopy, TEM). The thickness of the mercaptopropionic acid (MPA)/octanethiol (OT) layer was evaluated to be ∼1 nm, and, according to the authors, the stripe periodicity was 1 nm (see Table S1, Supporting Information, Jackson et al.). The simple geometrical model above therefore applies (3.8 nm + 2x1 nm, i.e., a 5.8 nm-diameter sphere with a 1 nm periodicity) and a strong dependence of the observed stripe width in the STM image is expected (1 nm, 0.9 nm, 0.6 nm, 0.3 nm). This is, however, not what is observed: experimentally, the apparent stripe width does not decrease as the tip moves away from the top of the hemisphere (Figure 1d). The stripe width is constant within ±10% (Figure 1e) and other particles have the same characteristics (Figure S1 and Table S1 of our SI). This discrepancy between the geometrical prediction and the experimental results cannot be explained by size polydispersity or a small error in particle sizing: for a particle 20% larger, the geometrical effect would still be very pronounced (1, 0.9, 0.8, 0.6, 0.4, 0.1 nm). The interpretation of Jackson et al.’s STM images as indicating the presence of regularly spaced stripes on the nanoparticles conflicts with geometry: if stripes are regularly spaced in 3D, they cannot be regularly spaced in 2D.

Another characteristic of the stripes is that they are aligned perpendicular to the scanning direction (additional discussion in our Supporting Information). Jackson et al. indicate that the existence of the stripes is confirmed by X-ray diffraction and TEM, however, the evidence provided is inconclusive (additional discussion in our Supporting Information).

The dependence (or not) of the stripe periodicity on experimental factors such as scanning speed,2a, 2k and on preparative factors, such as monolayer composition,^1^ has been the focus of several of the articles of the series. Direct measurement of the stripe width on the image was used, but this can introduce bias and experimental errors. To characterize regular patterns in noisy images, an attractive approach involves the computation of fast-Fourier transform power spectra images (FFT). FFT is analogous to electron diffraction patterns, and it can be used to analyze imperfect periodic structures and extract periodicities obscured by noise. For a perfect sinusoidal stripy image, the FFT image displays two peaks and their position is characteristic of the orientation and periodicity of the stripe, while for an image with defects and variations in stripe orientations, the FFT image shows arcs instead of peaks and the length of the arc and variation in brightness along the angle characterize the preferred orientation.^8^ **Figure** **2**a shows an STM image adapted from Jackson et al. The FFT of this image displays two marked lateral maxima corresponding to the stripes (Figure 2b). These maxima can be precisely located. As shown in the intensity profile, the periodicity measured along the *x*-axis of the FFT image is 1.3 ± 0.5 nm, instead of the 0.9 ± 0.1 nm or 1 nm reported for these particles.^1^ More importantly, the shape of the maxima in the FFT image is elongated and exactly aligned with the vertical axis. Two vertical lines going through these maxima can be observed. A vertical line in an FFT image reflects a series of modes in the real image that have exactly the same periodicity along the *x*-axis, but different periodicity along the *y*-axis, i.e., different wavelengths. Such modes cannot possibly be related to physical features of the sample because they have a defined wavelength, *λ*_x_, along the fast scanning axis, but no defined *λ*_y_ wavelength and therefore no overall wavelength *λ* (Figure 2b). For comparison, a model theoretical image and its FFT are presented (Figure 2d–f). The image was generated by pasting several times the theoretical image of Figure 1b with small rotations between –5 and +5 degrees. Two weak maxima can be seen on both sides. In spite of the near-perfect alignment of the theoretical stripy particles, the semi-circular shape of the maxima can be clearly seen and the wavelength of the corresponding modes is ∼0.9 nm. The stripy patterns observed in the images of Jackson et al. are reminiscent of feedback artifacts which generate high frequency periodic noise in scanning probe microscopy images.^9^ These can occur throughout an STM image or be localized to features with steep slopes due to mechanical perturbations of the tip. The presence of such oscillatory noise in the images of the stripy nanoparticles is in fact accepted by Jackson et al., although they argue that the noise is present on the substrate but not on the steep features (nanoparticles).^10^ It is likely that the oscillations are provoked by feedback artifacts modulated by the highly corrugated surface. Such mechanisms would be dependent on tip–nanoparticle interactions, which would certainly be affected by the monolayer composition. A close inspection of the STM image suggests that the phase of the oscillations is sometime reset at the beginning of a new scanning line, or when the tip approaches a nanoparticle, resulting in consecutive lines appearing shifted in relation to one another. The presence of such phase shifts between consecutive scanning lines is a hallmark of all published images of “stripy” nanoparticles. Such shifts are even more evident if a band pass frequency filter is applied to the images to remove noise at lower and higher frequencies than the stripes (Figure S3).

**Figure 2:**
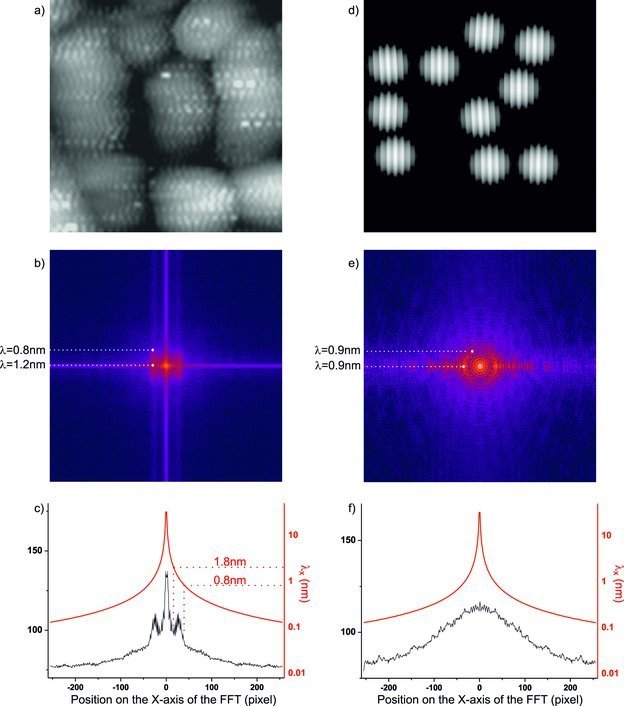
Fast-Fourier transform analysis of experimental and theoretical STM images. a) Experimental STM image (adapted from Figure 1a of Jackson et al. with permission.^1^ Copyrigh 2004, Macmillan Publishers Ltd.); b) FFT of the image shown in (a); c) average intensity profile of the entire FFT image along the *x*-axis; d) exemplary theoretical image of 10 nanoparticles; e) FFT of the image shown in (d); f) average intensity profile of the entire FFT image along the *x*-axis.

In 2008, the synthesis of water-soluble “stripy” nanoparticles using mixed layers of sodium 11-mercaptoundecanesulfonate (MUS) and OT was reported.2i In the same year, it was reported that these water-soluble “stripy” nanoparticles penetrate the plasma membrane of living cells without bilayer disruption and without being trapped in endosomes.2j Although both articles are founded on the claim that nanoparticles capped with 66% MUS and 33% OT are “stripy”, the only published evidence is a single STM image of a single particle. This image of a hydrophilic “stripy” nanoparticle is shown in **Figure** **3**a. The FFT (Figure 3b) has similar characteristics to the one shown in Figure 2b. By frequency filtering the image, the phase shifts between consecutive scanning lines become clearly apparent (Figure 3c,d). The stripe width is independent of the position of the tip over the nanoparticle (Figure 3e). This image cannot be the 2D representation of a sphere capped with regularly spaced stripes and the stripes cannot correspond to physical features of the sample.

**Figure 3:**
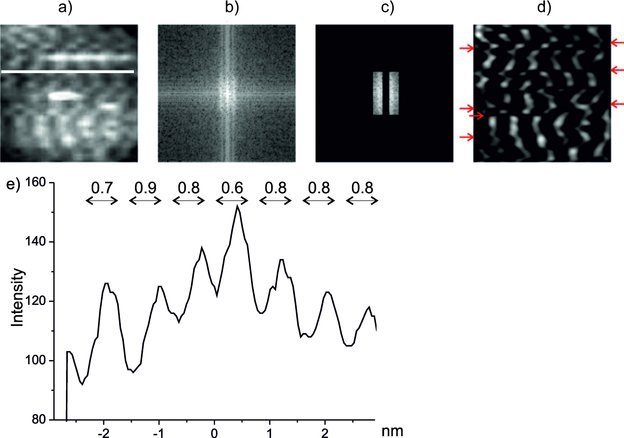
Water-soluble “stripy” (2:1 molar ratio of MUS:OT) nanoparticles. From left to right: a) STM image of a water-soluble particle (adapted from Uzun et al. with permission.2i Copyright 2008, The Royal Society of Chemistry); b) FFT of the image; c) frequency filter; d) Reverse FFT of (c), i.e., frequency-filtered image. Red arrows point to phase shifts between consecutive scanning lines. e) Intensity profile corresponding to the line in (a).

In the second part of the article, we now consider three unusual physicochemical properties of “stripy” nanoparticles which have been attributed to the nanoscale organization of the ligands into sub-nanometer stripes: the dependence of their colloidal stability on molecular composition, their interactions with proteins, and their uptake in live cells.

Based on visual observations of precipitation, Jackson et al. report that the dependence of the nanoparticle “solubility” in ethanol is non-monotonic as a function of monolayer composition, a property which is understood as being due to the formation of a unique surface structure.^1^ More quantitative data, with various solvents and nanoparticle compositions, are presented by the same group in a recent article by Centrone et al.2f Measurements done on the same system—i.e., gold nanoparticles capped with a mixed OT/MPA monolayer—in 2004 and 2008 give contradictory results (Figure S4). The origin of this discrepancy is not discussed in the more recent manuscript, and the range of saturation concentration values reported is surprisingly low and conflicts with our own measurements (additional discussion and Figure S2 in the Supporting Information).

Jackson et al. claim that the OT/MPA nanoparticles, because of their nanometer-sized stripes, are resistant to nonspecific protein adsorption and outperform traditional protein-repellent surfaces.^1^ This is repeated in several papers of the series,2a, 2d–f, 2h–k with a reference to Jackson et al.,^1^ but to our knowledge no data have been published on the interaction of OT/MPA nanoparticles with proteins. If confirmed by experimental evidence, this observation would be interesting because most protein-repellent materials, e.g., polyethylene glycol (PEG) or dextran, tend to have relatively hydrophilic surfaces, whereas the OT/MPA nanoparticles are not water-soluble.

Verma et al. report that MUS/OT nanoparticles have the ability to cross the cell membrane of living cells.2j This is an extremely interesting observation since nanomaterials normally enter cells by endocytosis and end up trapped in endosomes. This observation would therefore have immediate implications for nanoparticle-based sensing, imaging, and gene/drug delivery.6b In the original report, the largest body of evidence to support their interpretation consists of a confocal fluorescence study with limited additional electron microscopy.2j Fluorescence has, however, major drawbacks because gold nanoparticles larger than 2 nm are not intrinsically fluorescent^11^ and it is therefore necessary to include a fluorescently labeled thiolate in the monolayer. The latter is likely to have an effect on the organization of the monolayer. Furthermore, gold is an excellent fluorescence quencher and if the fluorescent label is released inside the cell, this would result in a strong increase of fluorescence, as well as a potential loss of co-localization between the observed fluorophore and the gold core. Loss of capping ligands by ligand exchange with intracellular thiol-containing biomolecules such as glutathione has been previously reported,^12^ and ligand exchange is strongly dependent on the nature and organization of the monolayer.^13^ In addition, although the penetration of the cell membrane without bilayer disruption by stripy nanoparticles is proposed as a general phenomenon, the dendritic cell line used by Verma et al.,2j DC2.4, has peculiar intracellular transport mechanisms: it has been shown that exogenous antigens added to DC2.4 cells accumulate in the endoplasmic reticulum and late endosomes, followed by retrograde transport to the cytoplasm.^14^ The various points made above makes the interpretation of the results reported by Verma et al. particularly difficult. Verma et al. also report that their results were confirmed in another cell line (MEFs), but the only evidence provided consists of one confocal fluorescence image of one cell (for each of the 4 types of nanoparticles).

To simplify this problem and evaluate whether MUS/OT-capped nanoparticles have the general properties of being able to cross the cell membrane, we decided to look at the delivery of MUS/OT nanoparticles into HeLa cells using photothermal microscopy.6b, 15 This microscopy technique is based on the large absorption cross-section of the gold core. It is immune from background scattering and does not require the inclusion of a fluorophore. The method is ultrasensitive, allowing the detection of single nanoparticles as small as 1.4 nm.15a The photothermal intensity is proportional to the nanoparticle volume and to the number of nanoparticles in the laser spot. Nanoparticles capped with the following composition were prepared as described by Verma et al.: 100% MUS, 66% MUS–33% OT, 33% MUS–66% OT (TEM and size distributions are shown in our SI, Figure S6). Quantitative measurements of salt-induced aggregation show that these materials have a relatively poor colloidal stability with a tendency to aggregate at moderate ionic strength, increasing with the proportion of OT in the monolayer (SI, Figure S7). The latter observation is unsurprising since OT is a hydrophobic ligand. Following a protocol identical to Verma et al., the nanoparticles were mixed in fresh cell medium immediately before being added to the cells, and incubated for 3 h. They were then rinsed, fixed, and imaged by photothermal and bright-field microscopy. As shown in **Figure** **4**, under the conditions used for this experiment, the photothermal background from the cells is negligible. For all three samples, nanoparticle uptake is observed with a pattern characteristic of endocytosis. Importantly, there is no significant difference of nanoparticle localization between cells exposed to nanoparticles capped with mixed versus homogeneous layers of thiolates. Photothermal microscopy has a very large dynamic range that is not easily represented on an image; to better show the intensity contrast between the high-intensity zones (endosomes) and the cytoplasm/nucleus/background, intensity profiles are shown for each image. This representation confirms unambiguously that no significant cytosolic localization occurs in HeLa cells for any of the tested samples. The zones of high photothermal signal, i.e., of high nanoparticle density, have intensities which are several orders of magnitude higher than the basal level. While these data do not contradict the observations made by Verma et al. (since another cell line was used), they show clearly that MUS/OT-capped nanoparticles do not have the general property of being able to cross the cell membrane.

**Figure 4:**
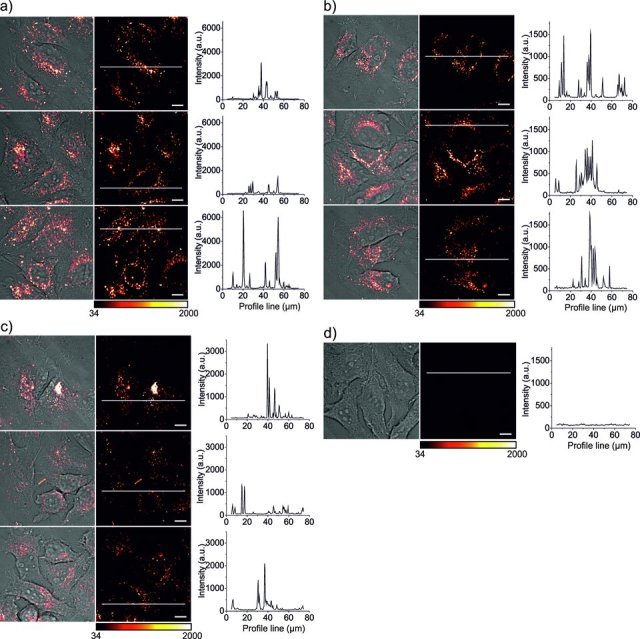
Internalization of a) 100% MUS-, b) 66% MUS–33%OT-, and, c) 33% MUS–66% OT-capped nanoparticles in HeLa cells. From left to right: overlay of bright field and photothermal image, photothermal image, and 1D profile section of the image (indicated by the white line on the photothermal image). The scale bars represent 10 μm.

We have revisited the evidence for the existence of stripes in mixed layers of alkane thiols on gold nanoparticles. The regular patterns observed in the STM images cannot correspond to regularly spaced stripes at the surface of nanoparticles, because if stripes are regularly spaced in 3D they cannot be in a 2D projection. Fast-Fourier transform analysis of the STM images indicates that the stripes are a scanning artifact. While our analysis suggests that the evidence for stripy nanoparticles is inconclusive, it certainly does not rule out the possibility of self-organization in mixed self-assembled monolayers. We observe that the range of saturation concentration values reported are surprising and conflict with our own measurements. Finally, we have examined the ability of “stripy nanoparticles” to cross the cell membrane by a careful photothermal examination of the localization of MUS/OT nanoparticles after uptake by HeLa cells, which indicates endosomal localization for all monolayer compositions.

## Experimental Section

*Image Processing:* STM images were copied from the original article's PDF (shown as grayscale images in Figure 2a, Figure 3a, and Figure S3). ImageJ was used for FFT processing. The contrast and brightness of the frequency-filtered images was automatically adjusted. There was no other processing or alteration of the images.

*Nanoparticle Synthesis:* A saturated solution of NaBH_4_ in HPLC-grade ethanol was prepared, and left stirring at 4 °C for 30 min. Meanwhile HAuCl_4_ (32 μm) was dissolved in HPLC-grade ethanol (18 mL) and left to stir for 10 min. The ligand or ligand mixture (32 μm) was added to the gold salt mixture and left stirring for another 10 min. To this gold and ligand mixture, the NaBH_4_ solution (7 mL) was slowly added dropwise. The solution turned from yellow, to orange, to brown, to a very dark brown. This mixture was left for a further 2 h stirring to complete the reaction. The reaction was then transferred to the freezer to aid precipitation of the particles. The supernatant was removed, and the particles were resuspended in ethanol (10 mL) three times to wash away excess ligand. The absence of excess ligands was confirmed by NMR.

*Solubility Study:* 100% OT particles (5 mg) were suspended in tetrahydrofuran (THF) (500 μL) and left undisturbed for 3 weeks. The concentration of nanoparticles was measured from the absorbance at 506 nm using an extinction coefficient *ε* = 3.64 × 10^6^ m^−1^ cm^−1^. The particles were first diluted in order to obtain a suitable absorbance (*A*_506_ < 2). The extinction coefficient was obtained (as Centrone et al.) from Liu et al.^16^

*Cell Culture and Nanoparticle Delivery:* HeLa cells were grown in Dulbecco's modified Eagle Medium (DMEM) supplemented with 10% fetal calf serum (FCS) (v/v) and 1% non-essential amino acids (v/v), at 37 °C, 5% CO_2_. Cells (between passages 8 to 20) were plated at 1 × 10^5^ cells/mL. For all experiments, nanoparticles (33% MUS, 66% MUS, or 100% MUS) were mixed with the complete medium (containing 10% FCS) and added immediately to the cells. The cells were incubated for 3 h (37 °C, 5% CO_2_) with nanoparticles at a final concentration of 400 nm. The cells were then washed three times with phosphate buffered saline (PBS), fixed with 4% paraformaldehyde/PBS (1 mL) for 15 min at room temperature, and then washed three times with PBS before adding ultra-pure water (2 mL). Fixed cells were stored at 4 °C until imaged by photothermal microscopy.

*Photothermal Microscopy:* The cells were fixed and observed by photothermal heterodyne microscopy. The absorbing beam (523 nm, Nd:YLF frequency-doubled laser) modulated at the frequency *ω* (*ω*/2π = 692.5 kHz) by an acousto-optic modulator and the probe beam (632.8 nm, HeNe laser) were focused on the sample using a Zeiss Achroplan 50 ×/0.9 NA oil immersion objective. The beam intensities were, respectively, 0.44 mW and 10.65 mW. The forward interfering fields were collected with a Zeiss Achroplan 40 ×/0.8 NA water dipping objective and sent onto a photodiode. The beat signal at the frequency *ω* was extracted via a lock-in amplifier and integrated over 10 ms. All images were completed by moving the sample with a piezo-electric device (Physik Instrumente) over the fixed laser beams and were taken at the altitude of 1 μm above the glass coverslip surface.

## Supplementary material

Detailed facts of importance to specialist readers are published as ”Supporting Information”. Such documents are peer-reviewed, but not copy-edited or typeset. They are made available as submitted by the authors.
