# Supplementary material for: Stripy Nanoparticles Revisited
Source: Small. 2012 Nov 23;8(24):3714–9. doi: 10.1002/smll.201001465 (PMC3561705; doi:10.1002/smll.201001465)
Supplement: Supplementary file 1 [file smll0008-3714-SD1.pdf]

Copyright WILEY-VCH Verlag GmbH & Co. KGaA, 69469 Weinheim, Germany, 2012.

Supporting Information

for *Small*, DOI: 10.1002/smll.201001465

Stripy Nanoparticles Revisited

*Yann Cesbron, Chris P. Shaw, James P. Birchall, Paul Free, and Raphaël Lévy**

Supplementary Information

Stripy Nanoparticles Revisited

*Yann Cesbron,‡ Chris P Shaw,‡ James P Birchall, Paul Free and Raphaël Lévy**

[*] Dr. R. Lévy, Dr. Y. Cesbron, Dr. C. P. Shaw, J. P. Birchall, Dr. P. Free
Structural and Chemical Biology, Institute of Integrative Biology, University of Liverpool
Biosciences Bldg, Crown Street, L69 7ZB (UK)
E-mail: rapha@liverpool.ac.uk
Dr. Y. Cesbron
CNRS, UMR 6290, Institut Génétique et Développement de Rennes, F-35043 Rennes, France; Université de Rennes 1, Université Européenne de Bretagne, Structure fédérative de recherche Biosit , Faculté de Médecine, F-35043 Rennes (France)
Dr. P. Free
Institute of Materials Research and Engineering, A*STAR (Agency for Science, Technology and Research), 3 Research Link, Singapore 117602 (Singapore)

[**] The authors thank BBSRC for a David Phillips Fellowship (BB/D020638/1, to R.L.) and Heike Arnolds for critical reading of the ms.

‡ Both authors contributed equally to this work

1. Alignment of stripes perpendicular to the scanning direction (additional discussion).

If we accept that these stripes are related to self-organized structures, then an argument is required to explain why all stripes (in the figure 1 of Jackson *et al.* reproduced below, Figure S1) are aligned in the same direction and why the latter direction happens to be perpendicular to the scanning direction. It was initially claimed that interdigitation forces the parallel alignment of stripes. This interpretation was based on data (not shown in the original article) indicating a loss of this alignment when the sample is prepared above the interdigitation temperature (legend of figure 1, p331[1]). In 2008, Hu *et al.* confirm the key role of interdigitation in directing the interactions between “stripy” nanoparticles.[2] However, the same year, the same group argued that interdigitation is energetically negligible in OT/MPA

(and others) stripy nanoparticles.[3] Centrone *et al.* write[3] (p9888): "[...] *because of the charges present in the NPs ligand shell, we can assume that the amount of interdigitation is negligible. We confirmed this using differential scanning calorimetry according to methods in the literature*". Whatever its role and extent in this system, interdigitation does not constitute a reasonable explanation for the observed alignment. While it could explain some degree of correlation between nanoparticles in direct contact, it fails to explain the perfect alignment of nanoparticles, which are separated by several others.

In a later publication, Jackson *et al.* explained that the stripes can only be visualized when the scanning direction is perpendicular to the stripes because of convolution with the STM tip.[4] This argument may explain why no stripes are observed parallel to the scanning direction, but it does not explain why stripes with a 70 to 80-degree angle to the scanning direction are not observed. It also fails to explain the correlation between the orientations of the nanoparticles. In addition, if we admit, for the sake of argument, that stripes can be observed only if they are within ± 5 degrees of the perpendicular of the scanning direction, then such observation should be a rare event. Only one in around 18 images should appear stripy (or one in 18 particles if there was no correlation between particles) whereas Jackson *et al.* report that the ripples were systematically observed[1] (p333, legend of figure 1): "*First, the imaging was performed on particles synthesised on separate occasions; each particle batch was cast on different substrates and imaged with multiple tips on various days over a period of months. The resulting images all showed the same rippled nanoparticles with a variation in the peak-to-peak spacing of less then 10%.*"

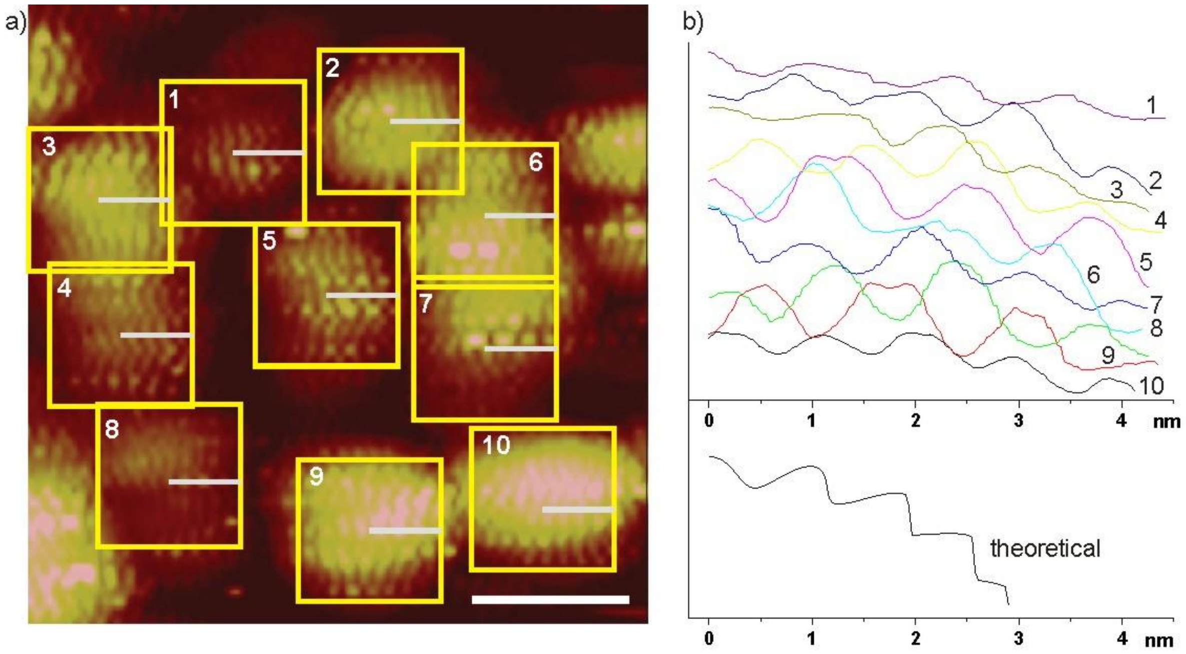

Figure S1: Height profiles of 10 nanoparticles observed by Jackson *et al.*[1] a) STM image reproduced from the figure 1a of Jackson *et al*. 10 nanoparticles are numbered and selected in the yellow squares; b) Top: height profiles of nanoparticles 1-10 (white lines in a); bottom: theoretical height profile for a 1 nm stripe on a 5.8 nm nanoparticle.

	1st	2nd	3rd	4th
Theoretical NP	0.98 nm	0.86 nm	0.64 nm	0.34 nm
NP1	1.0 nm	1.3 nm	1.0 nm	
NP2	1.2 nm	0.9 nm	1.0 nm	
NP3	1.3 nm	1.0 nm	0.9 nm	0.9 nm
NP4	1.0 nm	1.0 nm	1.1 nm	
NP5	1.2 nm	1.4 nm	1.1 nm	

NP6	1.1 nm	1.2 nm	1.0 nm	
NP7	1.0 nm	1.0 nm	1.0 nm	1.0 nm
NP8	1.0 nm	1.2 nm	1.3 nm	
NP9	1.2 nm	1.3 nm	1.3 nm	
NP10	0.9 nm	0.9 nm	0.9 nm	1.0 nm

Table S1: Stripe widths of theoretical and experimental 5.8 nm diameter nanoparticles. NP1-10 refers to the particles and line profiles shown in Figure S1a (and Figure 2a).

2. XRD and TEM (additional discussion)

Jackson *et al*. write: "*Indeed, all of the XRD plots of the rippled nanoparticles described in this paper showed peaks at 2θ ranging from 2.5° to 13°. Some of the peaks were temperature dependent, as is expected for peaks due to inter-particle packing arrangements.*[5] *However, one or two peaks were temperature independent, pointing to periodic arrangements, with 0.5–2.5 nm spacing, on single nanoparticles (see Supplementary Information, Fig. S1). Such temperature-independent peaks were never observed in homo-ligand nanoparticles.*" [The reference number has been changed in the quote above in order to match the reference list of the present article] The published results of the XRD study are however limited to three curves corresponding to two samples, which do not include the 2:1 OT/MPA 3.8 nm particles analyzed by STM. The XRD results of the non-stripy nanoparticles (homogeneous layer) are not shown which renders any comparison impossible. The XRD spectra show a number of peaks that are not assigned. The presence of "temperature-independent" peaks in these spectra does not constitute *per se* a proof of the existence of stripes at the surface of the nanoparticles. On the basis of the published results, there is no particular reason to believe that these peaks are due to the ligand shell.

Jackson *et al.* claim that the existence of stripes is confirmed by TEM; they write: "*Additional confirmation of the presence of ordered phase-separated domains was provided by transmission electron microscopy (TEM) images. In fact, in these images (see Supplementary Information, Fig. S2) we have found that there is an observable ring around the nanoparticles' metallic cores consisting of discrete dots spaced ~0.5–0.6 nm.*" The published results of the TEM study are however limited to one high-resolution image of one nanoparticle. The dots (attributed to ions bound to the stripes) are not really distinguishable from the variations of contrast due to the carbon grid. The TEM images of the non-stripy nanoparticles (homogeneous layer) are not shown which makes any comparison impossible. No conclusion regarding the structure of the capping layer can be drawn from this image.

3. Saturation concentrations (additional discussion)

Centrone *et al*. article is based on the measurement of saturation concentration of particles capped with various homogeneous and mixed monolayers.[3] The saturation concentration of particles capped with a homogeneous layer of octane thiol (100% OT, not stripy) is ~400 nM in five different solvents (Benzene, THF, Hexane, DCB and Chloroform). This value of 400 nM appears to be a maximum saturation value: none of the tested particles have a saturation concentration higher than 470 nM in any solvent. This is unexpected for two reasons. The first is that the solvent-nanoparticle interfacial energy, and therefore the saturation concentration, should vary significantly between these various solvents. The second reason is that 400 nM is remarkably low for a saturation concentration of colloids in a good solvent: it corresponds to a volume fraction equal to ~0.003% (0.0027% for a 6 nm diameter nanoparticle at a 400 nM concentration). For colloidal systems in a good solvent, volume fractions of 30 or 40% are routinely obtained (the absolute physical limit being in fact close packing of the spheres). We therefore decided to repeat Centrone *et al.* experiment for

nanoparticles capped with 100% OT in THF (TEM of nanoparticles and size distribution, Figure S5). Following the exact same procedure as Centrone *et al.*, we have been unable to measure a saturation concentration because equilibrium between a solid and a liquid phase was not observed even after 3 weeks: all the nanoparticles were still in suspension (Figure S2). We can therefore state categorically that the saturation concentration of gold nanoparticles coated with OT (3.6 ± 0.6 nm diameter) is greater than 8 μM, i.e. at least eighteen times higher than the one reported by Centrone *et al*.

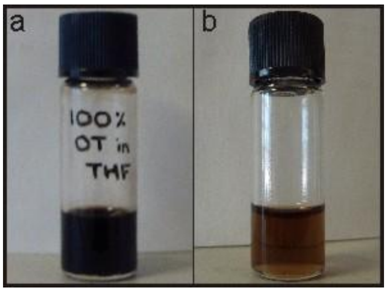

Figure S2. Photo of a 8 μM suspension of 3 nm diameter gold nanoparticles capped with OT three weeks after suspension in THF (a). A 450 nM suspension (reported as "saturation concentration" by Centrone *et al.*[3]) is shown for comparison (b).

3. Supporting figures

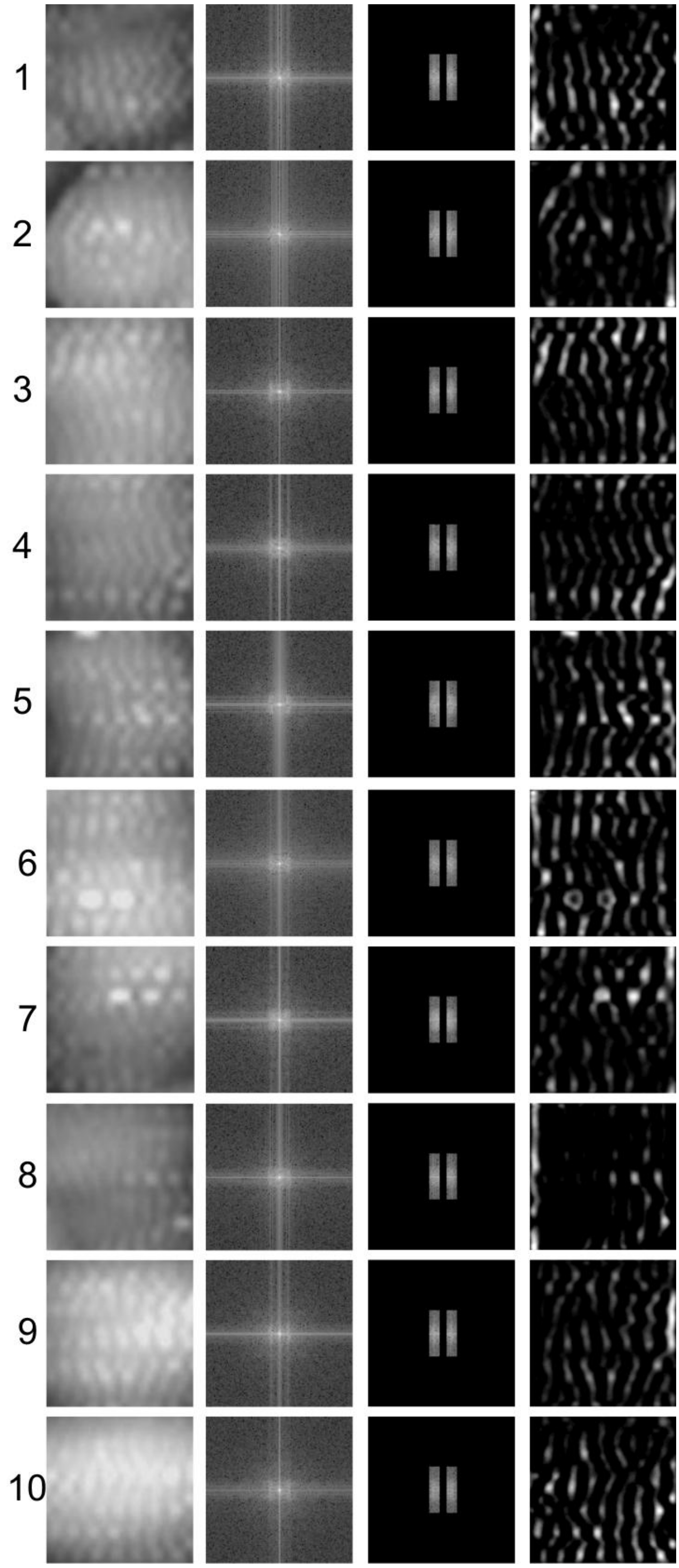

Figure S3: From left to right: individual particles cropped from the STM picture in Jackson *et al.*, 2004; FFT of the image; frequency filter; reverse FFT, i.e. frequency-filtered image.

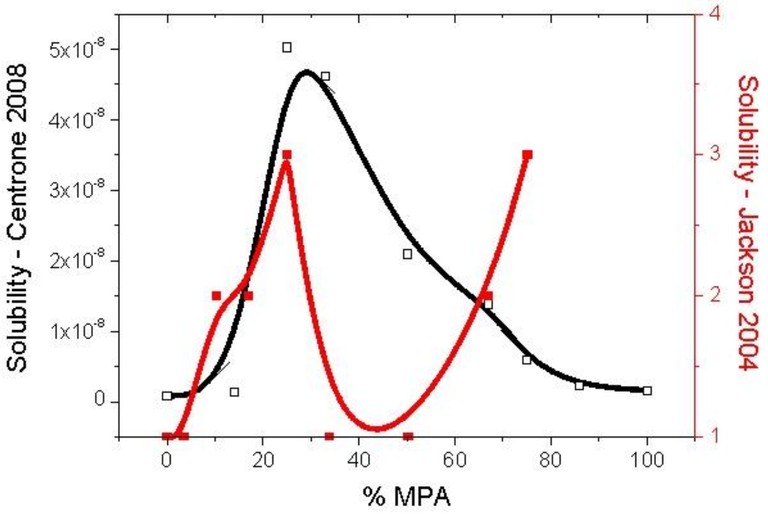

Figure S4: Comparison of the results reported by Centrone *et al*., 2008 (black curve, left Y axis) and by Jackson *et al*., 2004 (red curve, right Y axis) for solubility in ethanol; The solubility was measured as a saturation concentration expressed in molar in the 2008 study, while in the 2004 study, the solubility scale (right axis) was defined as follows: "*4 = highly soluble, that is, no precipitation visually observed, 3 = mostly soluble, that is, little precipitation observed over time with consequent slight decolouration of the solution; 2 = slightly soluble, that is, most of sample precipitated but a small coloration of the solution remains, 1 = totally insoluble.*" The data points are shown with symbols, black and red continuous lines (3-points splines) are shown to facilitate the visualization of the trends. The only difference between the two sets of experiments is that the gold core was synthesized in 2004 using a two-phase method while a one-phase method was used in 2008. This however should not affect the results given that, according to Jackson *et al.,* 2004, "*the ordering of the phase-domains is a result of a thermodynamic equilibrium, we prepared mixed-ligand nanoparticles in a two-step procedure starting from OT-coated nanoparticles and performing a place-exchange reaction with MPA. We observed ripples with the same spacing as those formed on nanoparticles synthesised in only one step. Because the same morphological end-point is reached from two different starting points, we conclude that the observed domains are an equilibrium state. This is additionally confirmed by the fact that the ripples on the nanoparticles are extremely stable; images of our samples taken over a period of more than eight months do not show any hint of change.*"

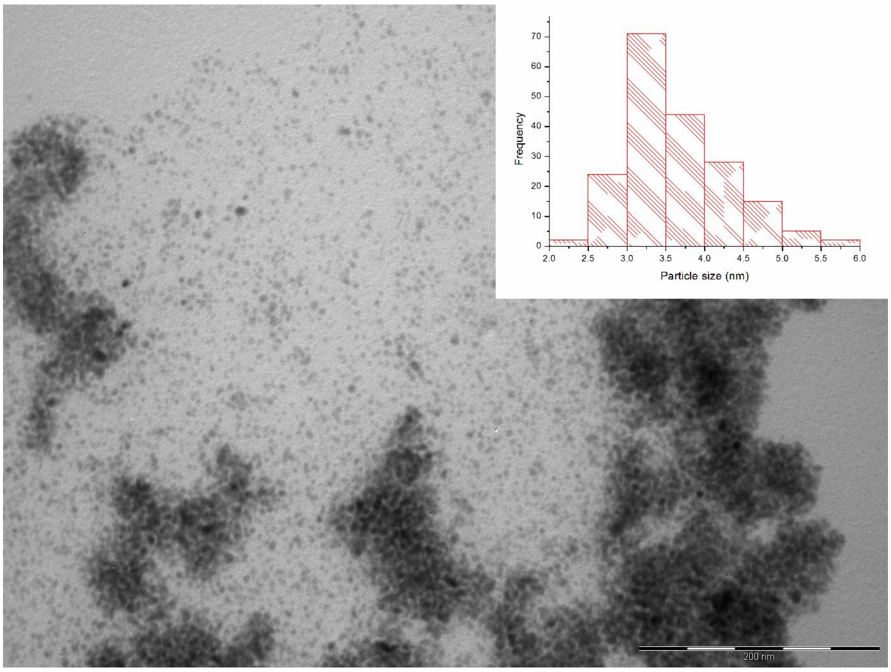

Figure S5: TEM micrograph and size distribution of gold nanoparticles capped with 100% OT.

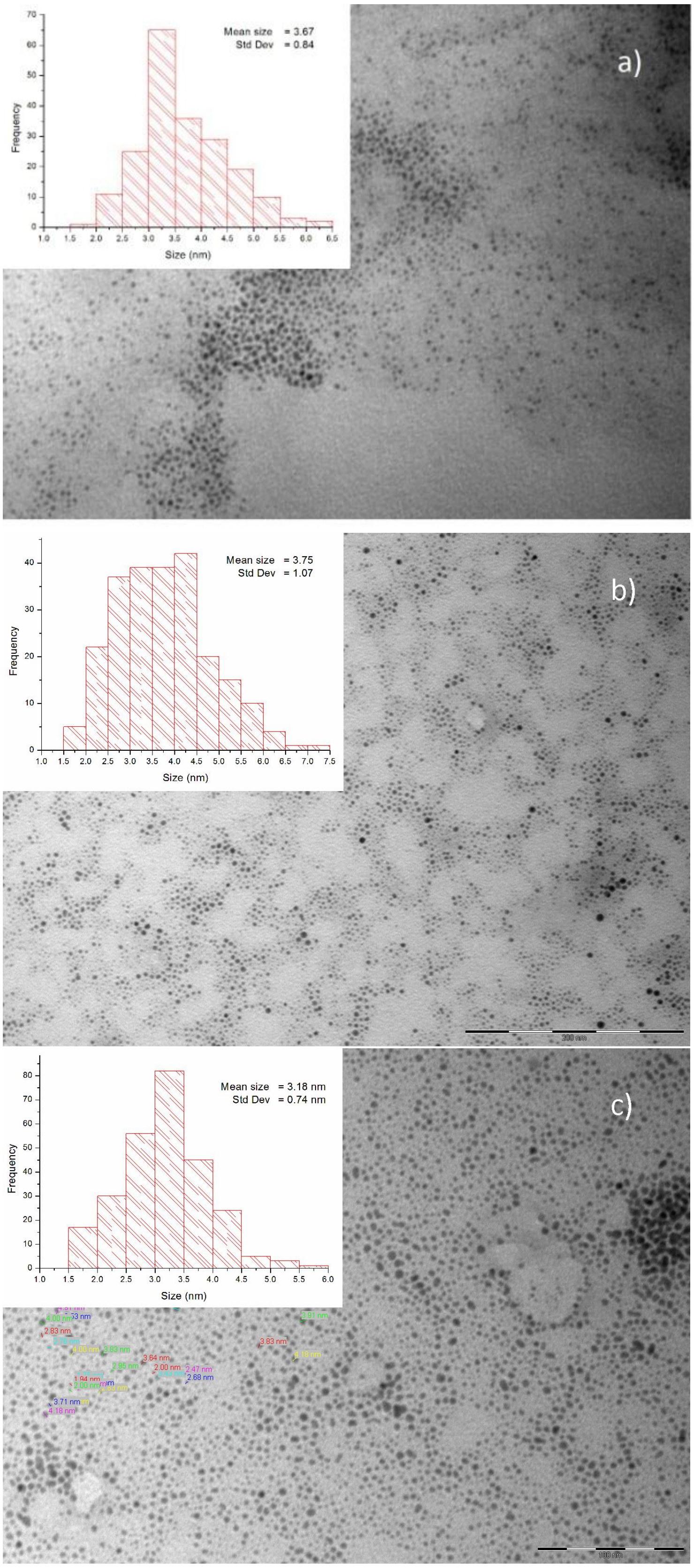

Figure S6: TEM micrographs and size distributions of gold nanoparticles with: a) MUS; b) 2:1 MUS:OT; c) 1:2 MUS:OT.

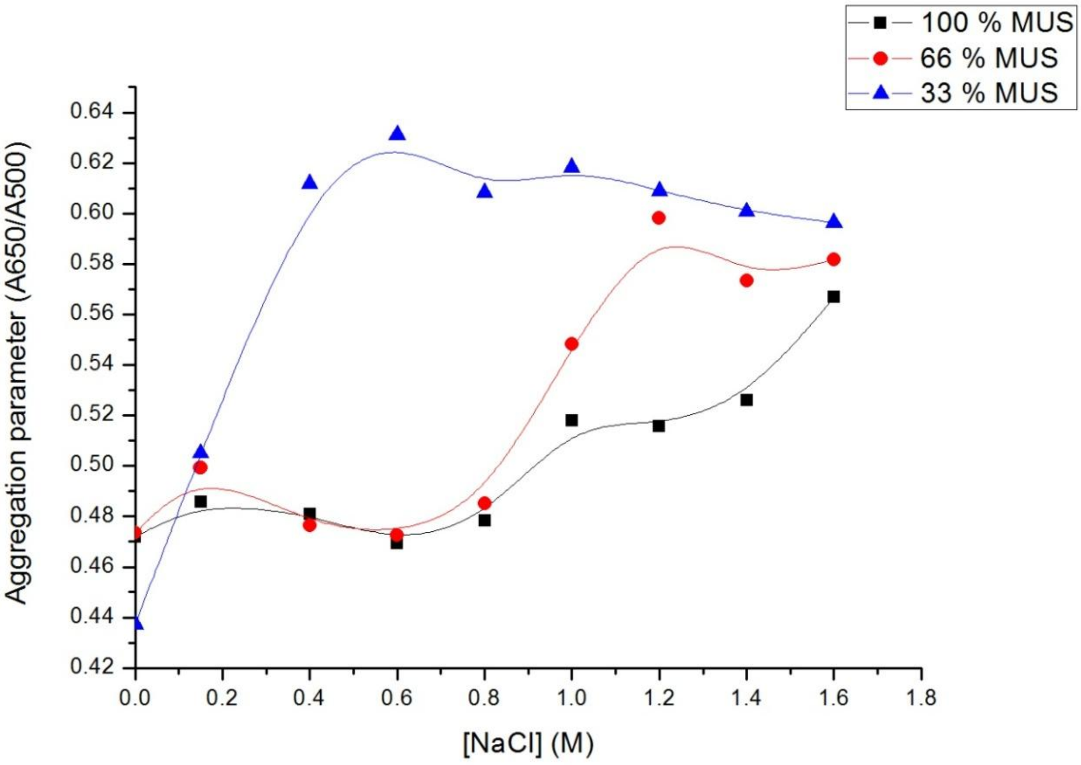

Figure S7: Colloidal stability of MUS/OT nanoparticles under increasing concentrations of NaCl. Blue – 2:1 OT:MUS; Red – 1:2 OT:MUS; Black – MUS.

REFERENCES

[1] A. M. Jackson, J. W. Myerson, F. Stellacci, *Nat. Mater.* **2004**, *3*, 330-336.
[2] Y. Hu, O. Uzun, C. Dubois, F. Stellacci, *J. Phys. Chem. C* **2008**, *112*, 6279-6284.
[3] A. Centrone, E. Penzo, M. Sharma, J. W. Myerson, A. M. Jackson, N. Marzari, F. Stellacci, *Proc. Natl. Acad. Sci. U. S. A.* **2008**, *105*, 9886-9891.
[4] A. M. Jackson, Y. Hu, P. J. Silva, F. Stellacci, *J. Am. Chem. Soc.* **2006**, *128*, 11135-11149.
[5] N. Sandhyarani, T. Pradeep, J. Chakrabarti, M. Yousuf, H. K. Sahu, *Phys. Rev. B: Condens. Matter Mater. Phys.* **2000**, *62*, R739-R742.
